# Knowledge, attitude, and practice towards knee osteoarthritis: a regional study in Chinese patients

**DOI:** 10.1007/s10067-025-07385-0

**Published:** 2025-03-11

**Authors:** Tao Liu, Chao Lin, Hui Shi, Qiang Ren, Xinmei Lin

**Affiliations:** 1 Department of Bone and Joint Surgery, Binzhou Medical University Hospital, No. 661 Huanghe 2 Road, Binzhou, 256603 Shandong China; 2 Department of Pediatric, Binzhou Medical University Hospital, No. 661 Huanghe 2 Road, Binzhou, 256603 Shandong China

**Keywords:** Attitude, Cross-sectional study, Knee osteoarthritis, Knowledge, Practice

## Abstract

**Introduction:**

Given the chronic nature of knee osteoarthritis and its reliance on self-management, patients’ knowledge, attitudes, and practice (KAP) significantly influence their ability to effectively manage knee osteoarthritis. This study aimed to investigate patients’ KAP towards knee osteoarthritis.

**Methods:**

This cross-sectional survey was conducted among patients with knee osteoarthritis, using a self-designed questionnaire.

**Results:**

A total of 764 (96.47%) valid questionnaires enrolled, including 451 (59.03%) females, with a mean age of 48.28 ± 7.08 years. Their mean KAP scores were 8.10 ± 3.67 (possible range 0–16), 26.40 ± 4.492 (possible range 9–45), and 20.67 ± 5.156 (possible range 7–35), respectively. Structural equation modelling showed that knowledge had direct effects on attitude and practice. Attitude was found to exert a direct impact on practice. Moreover, multivariate logistic regression showed that employment, family’s monthly income, duration of knee osteoarthritis, and medication were independently associated with knowledge. Age, sex, employment, and coachfellow were independently associated with attitude. Sex, employment, and marital status were independently associated with practice (all *P* < 0.05).

**Conclusion:**

Patients had inadequate knowledge, negative attitude, and inactive practice towards knee osteoarthritis. Age, gender, employment, and marital status, monthly income, duration of knee osteoarthritis, medication, and coachfellow might be associated with their KAP. It is recommended to enhance clinical practice through patient education, personalized exercise plans, and tailored care based on individual patient profiles for improved knee osteoarthritis management.

**Table:** 

**Key Points** • *Patients show inadequate KAP towards knee osteoarthritis.* •* Factors affecting KAP include age, gender, and income.* • *Need for patient education and personalized care in management.*

**Supplementary Information:**

The online version contains supplementary material available at 10.1007/s10067-025-07385-0.

## Introduction

Knee osteoarthritis, also known as degenerative knee osteoarthritis, is a prevalent form of osteoarthritis characterized by pain and a reduced health-related quality of life [1, 2]. This chronic inflammatory joint disease significantly hinders functionality and mobility, primarily affecting the knee joint and leading to substantial functional limitations and disability [3]. Patients with knee osteoarthritis commonly experience impaired knee function and severe pain. It is important to note that the management of knee pain is a critical treatment objective, as it not only plays a central role in addressing knee osteoarthritis but also predicts the radiographic progression [4]. In practical terms, knee osteoarthritis has a profound impact on patients’ overall quality of life and their ability to participate in work and daily activities [5].

Knowledge, attitude, and practice (KAP) surveys are often employed to explore behavioral practices alongside knowledge and risk perception. Previous studies have provided insights into various aspects of KAP related to osteoarthritis. For instance, a study conducted among physiotherapists in Nigeria found that although 81% had a fair knowledge of evidence-based therapeutic exercises, the majority (95.3%) exhibited a poor attitude towards implementing these interventions, highlighting a disconnect between knowledge and practice [6]. Another systematic review examined the impact of OA education on knowledge, beliefs, and behaviors, reporting trends of short-term knowledge improvement but limited changes in beliefs or behavior due to intrinsic and extrinsic factors influencing patient engagement and empowerment [7]. Similarly, a qualitative study exploring the self-management needs of older adults with knee OA revealed significant knowledge gaps and an urgent need for targeted educational strategies to enable effective self-management [8]. The scoping review by Ong et al. [9] revealed that while osteoarthritis (OA) patients demonstrated adequate knowledge about disease and exercise management, they lacked sufficient understanding of drug therapies and complementary strategies. Moreover, patient attitudes toward non-surgical interventions were found to be ambivalent, limiting their adherence to effective conservative managements, including physiotherapy and medications. These limitations highlight the need for further investigation into the factors influencing KAP to better inform interventions. The KAP model, a behavioral theory influencing human health behavior, emphasizes that individual behaviors are molded by their knowledge and attitudes. This model underscores the interplay among knowledge, attitudes, and behaviors in shaping health-related actions [10–12]. Given the chronic nature of knee osteoarthritis and its reliance on self-management, understanding patients’ knowledge, attitude, and practice towards this condition is imperative [13, 14]. Compared to prior studies that primarily focus on non-surgical interventions and conservative therapies, the present study employs a broader methodological framework and incorporates structural equation modeling to explore the direct and indirect pathways between KAP dimensions. This approach provides deeper insights into the relationships among knowledge, attitudes, and practices in knee osteoarthritis management, offering a novel contribution to the existing literature.

## Methods

### Study design and participants

This cross-sectional study was conducted between February and July 2023 at the Affiliated Hospital of Binzhou Medical College among knee osteoarthritis patients. Inclusion criteria encompassed patients formally diagnosed with knee osteoarthritis in a hospital setting. Exclusion criteria were as follows: (1) individuals with knee trauma, including injuries to the anterior and posterior cruciate ligaments, medial and lateral collateral ligaments, knee dislocation, patellar dislocation, and fractures around the knee; (2) those with other knee-related conditions such as meniscus tears, rheumatoid arthritis, gouty arthritis, pigmented villous nodular synovitis, and congenital knee deformities; (3) individuals with neurological diseases including cerebral infarction, cerebral hemorrhage, cervical spondylosis, lumbar intervertebral disc herniation, and lumbar spinal stenosis were also excluded from the study. This study was approved by the Ethic Committee of the Affiliated Hospital of Binzhou Medical College ([2023]-LSZ-(LW-156)), and all participants provided written informed consent.

### Questionnaire

The questionnaire design for this study was informed by the “Expert Consensus on Escalation Therapy of Knee Osteoarthritis (2018)” published by the Orthopedic Expert Committee of the Wu Jieping Medical Foundation and the Joint Surgery Group of the Orthopedics Branch of the Chinese Medical Association, along with the “Diagnosis and Treatment Guidelines for Osteoarthritis (2018)” issued by the Joint Surgery Group of the Orthopedics Branch of the Chinese Medical Association. To ensure the reliability of the questionnaire, a pilot study involving 34 participants was conducted, resulting in a favorable Cronbach’s α coefficient value of 0.884, signifying strong internal consistency for the questionnaire.

The final questionnaire (a version translated into English was attached as Appendix) comprised four dimensions of information collection, encompassing a total of 30 items. These dimensions consisted of basic information, which included 11 items: the knowledge dimension, which comprised 8 items; the attitude dimension, consisting of 6 items, with item 6 containing 4 sub-items; and the practice dimension, which included 5 items, with item 2 containing 2 sub-items, item 3 containing 3 sub-items, and item 4 containing 4 sub-items. In the knowledge dimension, each item had correct answers, and respondents were scored 2 points for being known well, 1 point for having heard of it, and 0 points for being unclear. The attitude and practice dimensions predominantly utilized a 5-point Likert scale to assess respondents’ sentiments and behaviors, with scores ranging from very positive (5 points) to very negative (1 point). For items 1 and 5 in the attitude dimension, 5 points were awarded for option a, 4 points for option b, 3 points for option c, 2 points for option d, and 1 point for option e. Conversely, items 2, 3, 4, and 6 in the attitude dimension were scored in the opposite order. In the practice dimension, items 1, 2, 3, and 5 were rated with 5 points for option a, 4 points for option b, 3 points for option c, 2 points for option d, and 1 point for option e. Item 4 in the practice dimension, which was descriptive in nature, did not receive any scoring. Achieving scores exceeding 70% of the maximum score in each section indicated adequate knowledge, a positive attitude, and proactive practice [15].

### Questionnaire distribution and quality control

The questionnaire distribution was conducted by a team comprising the five authors and two research assistants. Prior to distributing the questionnaires, the team members thoroughly familiarized themselves with the questionnaire’s content and its research objectives through collective discussions and study. During the distribution process, they effectively communicated the study’s purpose and content to the surveyed patients, addressed any inquiries in a comprehensive manner, and meticulously adhered to the established inclusion and exclusion criteria. The questionnaires were generated using the Questionnaire Star website and subsequently disseminated to the study participants within the outpatient clinic and inpatient wards of the Department of Bone and Joint Surgery at the Affiliated Hospital of Binzhou Medical College. Additionally, the questionnaires were shared with the participants through their respective WeChat groups. To ensure quality control, respondents were required to complete the questionnaire through WeChat login, limiting each IP address to one completed questionnaire. Questionnaires completed in less than 120 s, the option not following a normal logic, and any part of the KAP questionnaire selecting the same option were considered invalid questionnaires.

### Statistical analysis

Statistical analysis was conducted using SPSS 26.0 (IBM Corp., Armonk, N.Y., USA). Continuous variables were described using mean ± standard deviation (SD), and between-group comparisons were performed using *t*-tests or analysis of variance (ANOVA). Categorical variables were presented as *n* (%). Pearson correlation analysis was employed to assess the correlations between knowledge, attitude, and practice scores. In multivariate logistic regression analysis, achieving scores exceeding 70% of the maximum score was used as the cut-off value. Variables in univariate analysis with *P* < 0.05 were included in multivariate analysis. Structural equation modeling (SEM) was performed based on the following hypotheses: (1) knowledge has effect on attitude and practice; (2) attitude has effect on practice. Two-sided *P* < 0.05 were considered statistically significant.

## Results

A total of 792 questionnaires were collected. The following cases were excluded: those who chose the exact same option for one or all of the KAP dimensions were excluded; those who had an abnormal duration of diagnosis to date (in months) were excluded, and 764 cases of valid data remained, with a validity rate of 96.47%. Among them, 451 (59.03%) were female, with a mean age of 48.28 ± 7.08 years. Their mean KAP scores were 8.10 ± 3.67 (possible range 0–16), 26.40 ± 4.492 (possible range 9–45), and 20.67 ± 5.156 (possible range 7–35), respectively. The knowledge score varied from patients with different residence (*P* = 0.023), education (*P* < 0.001), employment (*P* < 0.001), family’s monthly per capita income (*P* < 0.001), type of medical insurance (*P* = 0.008), and medications (*P* < 0.001). As for the attitude score, there were difference among patients with different gender (*P* = 0.027) and employment (*P* = 0.020), and have coachfellow (*P* < 0.01). The difference of practice score were found among patients with different gender (*P* = 0.001), residence (*P* = 0.031), education (*P* < 0.001), employment (*P* < 0.001), family’s monthly per capita income (*P* = 0.002), marital status (*P* < 0.001), and medical insurance (*P* = 0.010) (Table 1).

**Table 1:** Demographic characteristics

Variables	*N*(%)	Knowledge, mean ± SD	*P*	Attitudes, mean ± SD	*P*	Practices, mean ± SD	*P*
Total	764(100)	8.10 ± 3.67		26.40 ± 4.492		20.67 ± 5.156	
Age (years), mean ± SD	48.28 ± 7.08						
Gender			0.479		0.027		0.001
Male	313(40.97)	8.26 ± 3.62		25.93 ± 4.33		21.41 ± 5.08	
Female	451(59.03)	7.98 ± 3.71		26.72 ± 4.57		20.16 ± 5.14	
Residence			0.023		0.219		0.031
Non-urban (rural/suburban)	349(45.68)	7.84 ± 3.89		26.20 ± 4.19		20.24 ± 5.23	
Urban	415(54.32)	8.31 ± 3.47		26.56 ± 4.72		21.04 ± 5.06	
Education			< 0.001		0.564		< 0.001
Middle school and below	222(29.06)	6.95 ± 3.76		26.61 ± 4.13		20.23 ± 4.94	
High school/technical secondary school	216(28.27)	8.27 ± 3.22		26.48 ± 4.55		20.38 ± 5.24	
Junior college	166(21.73)	8.34 ± 3.75		26.33 ± 4.49		20.36 ± 5.51	
Undergraduate and above	160(20.94)	9.19 ± 3.65		26.05 ± 4.88		22.00 ± 4.75	
Employment			< 0.001		0.020		< 0.001
Full-time	339(44.37)	8.94 ± 3.66		25.86 ± 4.77		21.89 ± 5.03	
Farming	122(15.97)	6.57 ± 3.60		26.80 ± 4.01		19.14 ± 5.45	
Jobless	303(39.66)	7.76 ± 3.46		26.83 ± 4.29		19.92 ± 4.86	
Family’s monthly per capita income, Yuan			< 0.001		0.353		0.002
< 2000	110(14.4)	6.18 ± 3.87		27.03 ± 4.23		19.33 ± 5.03	
2000–5000	325(42.54)	8.35 ± 3.36		26.31 ± 4.71		20.58 ± 5.24	
5000–10,000	256(33.51)	8.57 ± 3.68		26.44 ± 4.35		21.23 ± 4.92	
> 1000	73(9.55)	8.20 ± 3.86		25.68 ± 4.27		21.19 ± 5.39	
Marital status			0.144		0.222		< 0.001
Unmarried/divorced/widowed	27(3.53)	6.88 ± 4.44		27.37 ± 4.64		17.29 ± 6.15	
Married	737(96.47)	8.14 ± 3.64		26.36 ± 4.48		20.80 ± 5.07	
Have coachfellow			0.349		< 0.01		0.196
Yes	728(95.29)	8.11 ± 3.65		26.29 ± 4.47		20.71 ± 5.16	
No	36(4.71)	7.72 ± 4.09		28.44 ± 4.42		19.83 ± 4.95	
Type of medical insurance			0.008		0.240		0.010
Social medical insurance only	540(70.68)	8.23 ± 3.77		26.49 ± 4.43		20.40 ± 5.08	
Commercial medical insurance only	17(2.23)	6.82 ± 2.21		28.11 ± 3.05		19.70 ± 6.72	
Both of social and commercial medical insurance	193(25.26)	8.01 ± 3.46		26.03 ± 4.81		21.67 ± 4.98	
No medical insurance	14(1.83)	5.71 ± 3.22		25.71 ± 2.72		18.5 ± 6.50	
Duration of knee osteoarthritis/duration of first consultation (months)	27.71 ± 51.01	/	/	/	/	/	
Medication (glucosamine hydrochloride or glucosamine sulfate and chondroitin)			< 0.001		0.127		0.506
Yes	168(21.99)	9.54 ± 3.52		25.86 ± 4.30		20.66 ± 6.07	
No	596(78.01)	7.69 ± 3.61		26.55 ± 4.53		20.68 ± 4.87	

The distribution of knowledge dimensions revealed that the three questions with the highest number of participants choosing the “Known well” option were “Overweight can increase joint stress, so maintaining a standard body weight is important” (K5) with 26.83%, “Engaging in activities like cycling and swimming is beneficial, as they strengthen lower limb muscles, enhance joint stability, and help reduce weight” (K6) with 24.87%, and “In daily life, it’s important to wear soft, flexible athletic shoes, choose suitable insoles, and avoid wearing high-heeled shoes” (K8) with 23.82%. On the contrary, the three questions with the highest number of participants choosing the “Unclear” option were “Traditional therapy currently include physical therapy (such as heat therapy, transcutaneous electrical stimulation, and ultrasound), oral medications (glucosamine, nonsteroidal anti-inflammatory drugs, etc.), steroid injections, and sodium hyaluronate injections. In more severe cases of knee arthritis, arthroscopic surgery or artificial joint replacement surgery may be necessary” (K7) with 26.31%, “Osteoarthritis often occurs in the middle-aged and elderly population, with the prevalence increasing with age, and it may be more common in women than in men” (K3) with 23.17%, and “Knee osteoarthritis is very common, progresses slowly, and gradually develops symptoms over time, sometimes making it impossible to move when it becomes severe” (K1) with 19.11% (Table S1).

On patients’ attitudes towards knee osteoarthritis, a majority of patients (51.05%) strongly hope to receive more science education from healthcare professionals, while a substantial percentage (38.35%) express agreement with this sentiment (A1). Surprisingly, 72.51% do not believe that joint pain and limited joint mobility cause them to feel depressed (A2). When it comes to their confidence in treatment, 14.66% of patients feel that long and frequent hospital visits without relief have eroded their confidence (A3). Regarding their efforts to alleviate knee joint pain, a notable 34.29% of patients agree that they make their best effort to avoid walking and physical exercise (A4). Additionally, 55.89% of patients agree that exercise and dietary changes are necessary to address overweight-related issues, with 22.51% strongly agreeing (A5). When discussing barriers to exercise, a significant portion (23.95%) of patients either strongly agree or agree that a lack of exercise companions makes it challenging to adhere to exercise routines (A6.1). Furthermore, 15.84% of patients agree that concerns about increased pain after exercising are substantial barriers (A6.2). Time constraints are seen as barriers by 21.2% of patients (A6.3), and 22.77% of patients agree that the absence of suitable exercise facilities or venues poses a significant obstacle (A6.4) (Table S2).

In terms of practice dimension, strictly following medical advice was affirmed by 61.39% of the patients (P1). When it came to daily life management, a significant proportion of patients indicated the following behaviors: Avoiding excessive knee joint activities, such as prolonged walking or running, was practiced by 49.34% of patients (P2.1). Reducing activities like climbing stairs, prolonged standing, or kneeling positions was observed in 46.08% of patients (P2.2). In the realm of exercise, patients reported the following tendencies: Swimming as a form of exercise was adopted by 10.47% of patients (P3.1). Performing knee joint flexion and extension exercises without bearing weight was practiced by 17.8% of patients (P3.2). Consciously contracting the quadriceps muscles was undertaken by 14.18% of patients (P3.3). Patients’ willingness to accept various therapeutic approaches yielded the following results: Medication was accepted by 50.13% of patients (P4.1). Orthopedic device therapy found favor with 74.95% of patients (P4.2). Surgical therapy was considered by 80.5% of patients (P4.3). Appropriate physical therapy, including heat therapy, hydrotherapy, tui na, and acupuncture, was chosen by 47.9% of patients (P4.4). Lastly, the proactive pursuit of disease-related knowledge was reported by 34.56% of patients (P5) (Table S3).

**Fig. 1:**
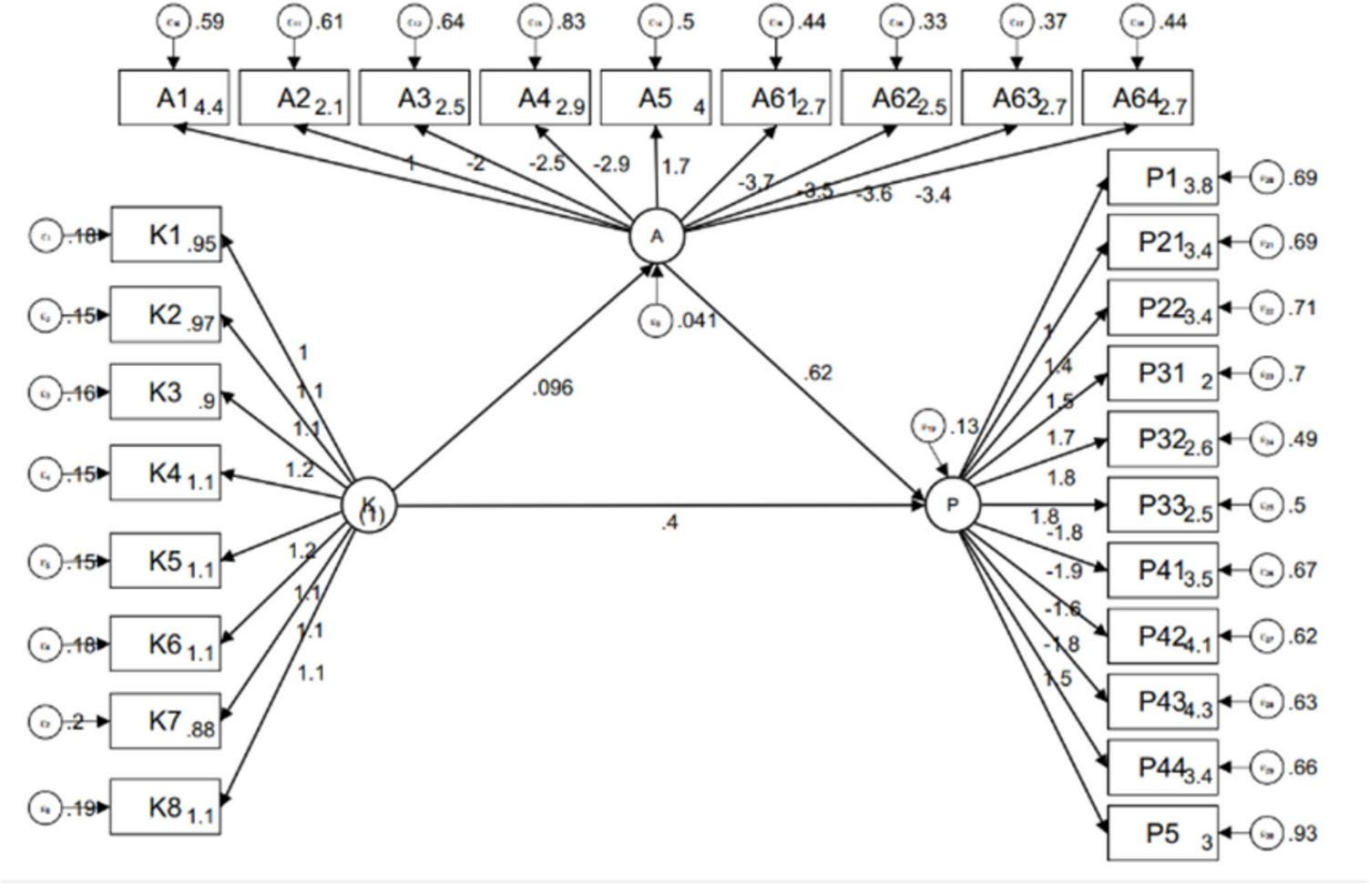
Structural equation modelling

Pearson’s correlation analysis showed that significant positive correlation was found between knowledge and practice (*r* = 0.3835, *P* < 0.001). Meanwhile, there were also negative correlations between knowledge and attitude (*r* =  − 0.0515, *P* = 0.1548), as well as attitude and practice (*r* =  − 0.2287, *P* < 0.001) (Table S4). The SEM demonstrate a highly favorable model fit indices, suggesting a well-fitting model (Table S5), and path analysis has shown that knowledge had direct effects on attitude (*β* = 0.096, *P* < 0.001) and practice (*β* = 0.400, *P* < 0.001). Additionally, attitude was found to exert a direct impact on practice (*β* = 0.618, *P* < 0.001) (Table 2 and Fig. 1). Further, mediation analysis showed similar effects, as evidenced by the presence of direct effects of knowledge on attitude (*β* = 0.09, *P* < 0.001), attitude on practice (*β* = 0.61, *P* < 0.001), and knowledge on practice (*β* = 0.039, *P* < 0.001), while knowledge also had an indirect effect on practice through attitude (*β* = 0.005, *P* < 0.001) (Table 3).

**Table 2:** Path analysis

			**Estimate**	**Std. err**	***P* > *z***
A	< –	K	0.096	0.026	< 0.001
P	< –	A	0.618	0.127	< 0.001
P	< –	K	0.400	0.053	< 0.001
K1	< –	K	1 (constrained)		
K2	< –	K	1.091	0.059	< 0.001
K3	< –	K	1.108	0.061	< 0.001
K4	< –	K	1.182	0.063	< 0.001
K5	< –	K	1.211	0.065	< 0.001
K6	< –	K	1.133	0.065	< 0.001
K7	< –	K	1.109	0.065	< 0.001
K8	< –	K	1.130	0.066	< 0.001
A1	< –	A	1 (constrained)		
A2	< –	A	− 1.971	0.316	< 0.001
A3	< –	A	− 2.535	0.397	< 0.001
A4	< –	A	− 2.864	0.455	< 0.001
A5	< –	A	1.665	0.270	< 0.001
A61	< –	A	− 3.739	0.567	< 0.001
A62	< –	A	− 3.492	0.526	< 0.001
A63	< –	A	− 3.573	0.544	< 0.001
A64	< –	A	− 3.441	0.528	< 0.001
P1	< –	P	1 (constrained)		
P21	< –	P	1.409	0.129	< 0.001
P22	< –	P	1.476	0.134	< 0.001
P31	< –	P	1.701	0.155	< 0.001
P32	< –	P	1.810	0.155	< 0.001
P33	< –	P	1.816	0.157	< 0.001
P41	< –	P	− 1.830	0.161	< 0.001
P42	< –	P	− 1.867	0.165	< 0.001
P43	< –	P	− 1.553	0.144	< 0.001
P44	< –	P	− 1.781	0.158	< 0.001
P5	< –	P	1.547	0.147	< 0.001

**Table 3:** Mediation analysis

**Model paths**		**Total effects**		**Direct effect**		**Indirect effect**	
		**β (95% CI)**	***P***	**β (95% CI)**	***P***	**β (95% CI)**	***P***
A sum < -							
	K sum	0.09(0.04,0.14)	< 0.001	0.09(0.04,0.14)	< 0.001		
P sum < -							
	A sum	0.61(0.36,0.86)	< 0.001	0.61(0.36,0.86)	< 0.001		
	K sum	0.45(0.34,0.57)	< 0.001	0.39(0.29,0.50)	< 0.001	0.05(0.02,0.09)	< 0.001

Multivariate logistic regression showed farming (OR = 0.442, 95% CI 0.232–0.842, *P* = 0.013), being jobless (OR = 0.648, 95% CI 0.435–0.965, *P* = 0.033), having 2000–5000 and 5000–10,000 of family’s monthly income (OR = 1.953, 95% CI 1.035–3.683, *P* = 0.039 and OR = 2.030, 95% CI 1.039–3.965, *P* = 0.038), duration of knee osteoarthritis (OR = 1.003, 95% CI 1.000–1.006, *P* = 0.012), and having not applied glucosamine hydrochloride or glucosamine sulfate and chondroitin (OR = 0.523, 95% CI 0.358–0.763, *P* = 0.001). Meanwhile, age (OR = 1.025, 95% CI 1.003–1.048, *P* = 0.026), being female (OR = 1.471, 95% CI 1.085–1.994, *P* = 0.013), farming (OR = 1.668, 95% CI 1.061–2.623, *P* = 0.027), being jobless (OR = 1.471, 95% CI 1.029–1.949, *P* = 0.032), and living alone (OR = 2.240, 95% CI 1.064–4.717, *P* = 0.034) were independently associated with attitude. Furthermore, knowledge (OR = 1.231, 95% CI 1.169–1.296, *P* < 0.001), attitude (OR = 0.922, 95% CI 0.889–0.957, *P* < 0.001), being female (OR = 0.680, 95% CI 0.486–0.952, *P* = 0.025), being jobless (OR = 0.647, 95% CI 0.441–0.950, *P* = 0.027), and being married (OR = 2.781, 95% CI 1.044–7.405, *P* = 0.041) were independently associated with practice (Table 4).

**Table 4:** Multivariate logistic regression analysis for knowledge, attitude, and practice

	**Univariate analysis**		**Multivariate analysis**	
	**OR (95%CI)**	***P***	**OR (95%CI)**	***P***
Knowledge				
Age (years), mean ± SD	1.013(0.991,1.036)	0.234		
Gender				
Male				
Female	0.988(0.713,1.368)	0.944		
Residence				
Non-urban (rural/suburban)				
Urban	1.119(0.810,1.546)	0.492		
Education				
Middle school and below				
High school/technical secondary school	1.092(0.694,1.718)	0.702	0.751(0.457,1.232)	0.258
Junior college	1.698(1.072,2.689)	0.024	0.993(0.583,1.691)	0.981
Undergraduate and above	2.004(1.268,3.167)	0.003	1.025(0.577,1.823)	0.931
Employment				
Full-time				
Farming	0.359(0.209,0.615)	< 0.001	0.442(0.232,0.842)	0.013
Jobless	0.585(0.412,0.829)	0.003	0.648(0.435,0.965)	0.033
Family’s monthly per capita income, Yuan				
< 2000				
2000–5000	2.425(1.336,4.403)	0.004	1.953(1.035,3.683)	0.039
5000–10,000	2.826(1.542,5.180)	0.001	2.030(1.039,3.965)	0.038
> 1000	2.389(1.129,5.054)	0.023	1.913(0.852,4.295)	0.116
Marital status				
Unmarried/divorced/widowed				
Married	1.627(0.608,4.356)	0.332		
Have coachfellow				
Yes				
No (live alone)	0.911(0.421,1.971)	0.813		
Type of medical insurance				
Social medical insurance only				
Commercial medical insurance only	0.334(0.075,1.478)	0.149		
Both of social and commercial medical insurance	0.806(0.552,1.177)	0.266		
No medical insurance	0.192(0.025,1.486)	0.114		
Duration of knee osteoarthritis/duration of first consultation (months)	1.003(1.000,1.006)	0.011	1.003(1.000,1.006)	0.012
Medication (glucosamine hydrochloride or glucosamine sulfate and chondroitin)				
Yes				
No	0.516(0.358,0.744)	< 0.001	0.523(0.358,0.763)	0.001
Attitude				
Knowledge	0.982(0.945,1.021)	0.367		
Age (years), mean ± SD	1.026(1.005,1.047)	0.013	1.025(1.003,1.048)	0.026
Gender				
Male				
Female	1.346(1.008,1.798)	0.044	1.471(1.085,1.994)	0.013
Residence				
Non-urban (rural/suburban)				
Urban	1.156(0.869,1.537)	0.318		
Education				
Middle school and below				
High school/technical secondary school	0.849(0.583,1.235)	0.393		
Junior college	0.924(0.618,1.383)	0.704		
Undergraduate and above	0.797(0.530,1.198)	0.276		
Employment				
Full-time				
Farming	1.836(1.206,2.793)	0.005	1.668(1.061,2.623)	0.027
Jobless	1.504(1.101,2.054)	0.01	1.417(1.029,1.949)	0.032
Family’s monthly per capita income, Yuan				
< 2000				
2000–5000	0.705(0.456,1.091)	0.117		
5000–10,000	0.794(0.506,1.245)	0.316		
> 1000	0.582(0.320,1.057)	0.076		
Marital status				
Unmarried/divorced/widowed				
Married	0.689(0.315,1.505)	0.351		
Have coachfellow				
Yes				
No (live alone)	2.323(1.126,4.791)	0.022	2.240(1.064,4.717)	0.034
Type of medical insurance				
Social medical insurance only				
Commercial medical insurance only	3.178(1.023,9.872)	0.045	2.787(0.880,8.817)	0.081
Both of social and commercial medical insurance	0.928(0.668,1.290)	0.659	1.035(0.737,1.453)	0.841
No medical insurance	0.543(0.179,1.642)	0.28	0.476(0.154,1.469)	0.197
Duration of knee osteoarthritis/duration of first consultation (months)	0.999(0.996,1.002)	0.852		
Medication (glucosamine hydrochloride or glucosamine sulfate and chondroitin)				
Yes				
No	1.263(0.896,1.781)	0.181		
Practice				
Knowledge	1.240(1.182,1.302)	< 0.001	1.231(1.169,1.296)	< 0.001
Attitude	0.921(0.892,0.952)	< 0.001	0.922(0.889,0.957)	< 0.001
Age (years), mean ± SD	1.000(0.981,1.021)	0.923	1.004(0.980,1.029)	0.713
Gender				
Male				
Female	0.675(0.505,0.903)	0.008	0.680(0.486,0.952)	0.025
Residence				
Non-urban (rural/suburban)				
Urban	1.331(1.000,1.770)	0.05	1.231(0.848,1.786)	0.274
Education				
Middle school and below				
High school/technical secondary school	1.219(0.837,1.775)	0.3	0.725(0.462,1.138)	0.163
Junior college	1.242(0.830,1.859)	0.291	0.637(0.383,1.062)	0.084
Undergraduate and above	2.307(1.517,3.508)	< 0.001	0.965(0.547,1.704)	0.904
Employment				
Full-time				
Farming	0.380(0.248,0.581)	< 0.001	0.598(0.328,1.089)	0.093
Jobless	0.487(0.355,0.668)	< 0.001	0.647(0.441,0.950)	0.027
Family’s monthly per capita income, Yuan				
< 2000				
2000–5000	2.046(1.308,3.200)	0.002	1.237(0.734,2.087)	0.424
5000–10,000	2.455(1.545,3.899)	< 0.001	1.269(0.719,2.241)	0.41
> 1000	1.676(0.917,3.064)	0.093	0.908(0.445,1.852)	0.792
Marital status				
Unmarried/divorced/widowed				
Married	3.142(1.312,7.520)	0.010	2.781(1.044,7.405)	0.041
Have coachfellow				
Yes				
No (live alone)	0.837(0.428,1.637)	0.604		
Type of medical insurance				
Social medical insurance only				
Commercial medical insurance only	0.888(0.337,2.338)	0.811		
Both of social and commercial medical insurance	1.325(0.951,1.845)	0.096		
No medical insurance	0.555(0.183,1.679)	0.298		
Duration of knee osteoarthritis/duration of first consultation (months)	1.000(0.997,1.003)	0.825		
Medication (glucosamine hydrochloride or glucosamine sulfate and chondroitin)				
Yes				
No		0.25		

## Discussion

Patients had inadequate knowledge, negative attitude, and inactive practice towards knee osteoarthritis. Age, gender, employment, and marital status, monthly income, duration of knee osteoarthritis, medication, and coachfellow might be associated with their KAP. These findings might be beneficial to enhance clinical practice through patient education, personalized exercise plans, and tailored care based on individual patient profiles for improved knee osteoarthritis management.

The findings of this study suggest that patients generally lack adequate knowledge, hold negative attitudes, and exhibit inactive practices with respect to knee osteoarthritis [16]. Addressing these trends is crucial for improving clinical practice [17]. Healthcare providers should prioritize patient education to enhance their understanding of knee osteoarthritis, its symptoms, progression, and treatment options [18]. Fostering a more positive outlook toward the condition can motivate patients to actively engage in their treatment plans, which should include regular exercise, weight management, and consistent adherence to recommended therapies [19]. These efforts are essential for effective knee osteoarthritis management and can lead to improved patient outcomes and greater overall satisfaction with their care [20].

Notably, knowledge scores varied across different demographic factors, such as residence, education, employment, family income, type of medical insurance, and medication use, underlining the need for tailored educational interventions to address these disparities [21]. Similarly, attitude scores exhibited differences based on gender, employment, and cohabitation status, underscoring the importance of addressing these factors when shaping patient-centered care and support strategies [22]. Moreover, practice scores differed among patients based on gender, residence, education, employment, family income, marital status, and type of medical insurance, highlighting the complex interplay of sociodemographic variables in shaping patients’ adherence to recommended practices for knee osteoarthritis management [23]. Multivariate logistic regression analysis further elucidated the independent associations between various factors and patient outcomes [24]. For instance, farming and joblessness were associated with both improved and reduced knowledge and attitude scores, respectively, while factors like family income, duration of diagnosis, and medication use demonstrated significant impacts on patients’ knowledge and practices. Additionally, age, gender, cohabitation status, and employment status were independently linked to patients’ attitudes, while knowledge, attitude, gender, joblessness, and marital status played a role in shaping patients’ practices. These comprehensive findings not only shed light on the multifaceted nature of patient behavior regarding knee osteoarthritis but also provide valuable insights for healthcare providers to develop targeted interventions and strategies for improving clinical practice and patient outcomes in this population [11, 25, 26].

The distribution of knowledge dimensions within the study provides valuable insights into patients’ understanding of knee osteoarthritis. Notably, participants exhibited a relatively high level of knowledge regarding certain aspects of the condition, with the majority expressing confidence in statements emphasizing the importance of maintaining a standard body weight, engaging in muscle-strengthening activities like cycling and swimming, and choosing appropriate footwear [27]. However, there were significant gaps in knowledge, as evidenced by the substantial proportion of participants finding certain statements unclear [28]. These included key details about traditional therapies, the demographic prevalence of knee osteoarthritis, and the progressive nature of the condition. To enhance clinical practice, it is imperative to address these knowledge gaps systematically. Healthcare providers should prioritize patient education efforts that focus on clarifying the areas of uncertainty, offering comprehensive information on treatment options, emphasizing the demographic factors associated with knee osteoarthritis, and elucidating the condition’s gradual onset and impact [29, 30]. By addressing these specific gaps in knowledge, healthcare professionals can empower patients with the information they need to make informed decisions and actively participate in their care, ultimately improving clinical outcomes and patient satisfaction [31, 32].

The attitudes of patients towards knee osteoarthritis, as revealed by this study, offer critical insights into their perspectives and preferences regarding healthcare delivery and self-management [33]. A significant proportion of patients express a strong desire for more comprehensive science education from healthcare professionals, underscoring the importance of patient-provider communication and knowledge dissemination [34]. Surprisingly, a notable majority do not associate joint pain and limited joint mobility with depression, suggesting a need for greater awareness of the psychological and emotional aspects of this condition [35, 36]. Furthermore, the erosion of confidence in treatment due to prolonged hospital visits without relief highlights the significance of timely and effective interventions. Patients’ efforts to alleviate knee joint pain vary, with a substantial portion actively avoiding physical activity, emphasizing the importance of tailored exercise regimens and pain management strategies [37]. The recognition of the necessity of exercise and dietary changes for addressing weight-related issues signifies an opportunity for healthcare providers to promote holistic lifestyle modifications [38]. Barriers to exercise, such as the absence of companions, concerns about increased pain, time constraints, and limited access to suitable facilities, pose significant challenges, necessitating targeted interventions to address these impediments [39]. To enhance clinical practice, it is imperative to acknowledge and address these patient attitudes systematically, tailoring interventions to improve communication, emotional support, confidence-building, and accessibility, ultimately fostering better patient engagement and outcomes in knee osteoarthritis management [32, 40, 41].

The results concerning patients’ practices in managing knee osteoarthritis underscore the diversity of approaches employed by individuals in their efforts to cope with the condition. Encouragingly, a significant majority of patients express their commitment to adhering strictly to medical advice, highlighting the potential for effective patient-provider collaboration in treatment plans [42, 43]. In daily life management, many patients actively engage in behaviors aimed at reducing excessive stress on their knee joints, such as avoiding prolonged walking or running, and minimizing activities involving knee strain, like climbing stairs or kneeling. These practices reflect a fundamental understanding of the importance of joint preservation. In the realm of exercise, while a portion of patients adopts low-impact activities like swimming and specific joint exercises, there is room for promoting broader adoption of therapeutic exercises that enhance joint stability and reduce pain. Patients also exhibit a willingness to explore diverse therapeutic approaches, including medication, orthopedic devices, surgical interventions, and physical therapies, reflecting their open-mindedness to various treatment modalities [44]. However, the uptake of disease-related knowledge remains relatively low, suggesting an opportunity for healthcare providers to emphasize the importance of patient education in empowering individuals to make informed decisions about their care. To improve clinical practice, it is imperative for healthcare providers to build on patients’ existing commitment to medical advice and healthy lifestyle practices while promoting a more comprehensive understanding of therapeutic options and the importance of self-education, ultimately fostering more effective patient engagement and better outcomes in knee osteoarthritis management [45, 46].

The correlation and SEM analyses provide valuable insights into the interplay of patients’ knowledge, attitude, and practice concerning knee osteoarthritis. Correlation analysis reveals a significant positive association between knowledge and practice, highlighting that a better understanding of the condition is linked to more proactive self-management [11, 26]. Conversely, there are negative correlations between knowledge and attitude, as well as attitude and practice, suggesting that while knowledge positively influences practice, it may not necessarily align with a positive attitude, and a positive attitude does not always translate into improved practice. The SEM results further confirm these relationships, demonstrating that knowledge directly influences both attitude and practice, emphasizing that interventions aiming to modify patient attitudes could be highly effective. Moreover, the mediation analysis confirmed that knowledge not only directly affects practice but also indirectly through its impact on attitudes, indicating the significance of fostering positive patient attitudes toward knee osteoarthritis management to drive more effective and proactive practices [47, 48]. The identified pathways in our study emphasize the need for multi-faceted interventions that do not solely focus on disseminating information but also on shaping attitudes. To enhance clinical practice, healthcare providers should prioritize comprehensive patient education initiatives that not only improve knowledge but also cultivate positive attitudes, recognizing that these factors together contribute to more favorable patient outcomes in knee osteoarthritis management.

The limitations of this study include its cross-sectional design, which limits the establishment of causal relationships between variables. The study was conducted at a single medical center, potentially limiting the generalizability of findings to broader populations. Data collection relied on self-report measures, introducing the possibility of recall bias and social desirability bias. Additionally, the study did not explore certain factors such as socioeconomic status or comorbidities that could influence knowledge, attitude, and practice regarding knee osteoarthritis. Future research could address these limitations by employing longitudinal designs, including diverse patient populations, and incorporating more comprehensive data collection methods.

In conclusion, patients had inadequate knowledge, negative attitude, and inactive practice towards knee osteoarthritis. Age, gender, employment, and marital status, monthly income, duration of knee osteoarthritis, medication, and coachfellow might be associated with their KAP. To enhance clinical practice for knee osteoarthritis patients, it is crucial to implement structured patient education programs to improve knowledge and foster positive attitudes. Personalized exercise regimens and support systems should be developed to address barriers to physical activity.

## Supplementary Information

Below is the link to the electronic supplementary material.

Supplementary Material 1 (DOCX 30.6 KB)

Supplementary Material 2 (DOCX 15.7 KB)

Supplementary Material 3 (DOCX 15.7 KB)

Supplementary Material 4 (DOCX 16.1 KB)

Supplementary Material 5 (DOCX 13.8 KB)

Supplementary Material 6 (DOCX 13.8 KB)

Supplementary Material 7 (DOCX 28.1 KB)

## Data Availability

All data generated or analyzed during this study are included in this article.
