# Supplementary material for: Knowledge, attitude, and practice towards knee osteoarthritis: a regional study in Chinese patients
Source: Clin Rheumatol. 2025 Mar 11;44(4):1819–30. doi: 10.1007/s10067-025-07385-0 (PMC11993439; doi:10.1007/s10067-025-07385-0)
Supplement: Supplementary file 2 — Supplementary Material 2 (DOCX 15.7 KB) [file 10067_2025_7385_MOESM2_ESM.docx]

**Table S1. Distribution of Knowledge Dimension**

|  | **N (%)** | | |
| --- | --- | --- | --- |
|  | **Known well** | **Heard of** | **Unclear** |
| 1. **Knee osteoarthritis is very common, progresses slowly, and gradually develops symptoms over time, sometimes making it impossible to move when it becomes severe.** | 108(14.14) | 510(66.75) | 146(19.11) |
| 1. **The treatment goals for knee osteoarthritis are to alleviate pain, slow down disease progression, correct deformities, improve or restore joint function, and enhance the quality of life.** | 117(15.31) | 509(66.62) | 138(18.06) |
| 1. **Osteoarthritis often occurs in the middle-aged and elderly population, with the prevalence increasing with age, and it may be more common in women than in men.** | 98(12.83) | 489(64.01) | 177(23.17) |
| 1. **Patients with knee osteoarthritis should avoid inappropriate exercise, pay attention to correcting poor posture, refrain from prolonged running, jumping, squatting, and try to avoid climbing stairs for prolonged periods.** | 165(21.6) | 484(63.35) | 115(15.05) |
| 1. **Overweight can increase joint stress, so maintaining a standard body weight is important.** | 205(26.83) | 466(60.99) | 93(12.17) |
| 1. **Engaging in activities like cycling and swimming is beneficial, as they strengthen lower limb muscles, enhance joint stability, and help reduce weight.** | 190(24.87) | 468(61.26) | 106(13.87) |
| 1. **Traditional therapy currently include physical therapy (such as heat therapy, transcutaneous electrical stimulation, and ultrasound), oral medications (glucosamine, nonsteroidal anti-inflammatory drugs, etc.), steroid injections, and sodium hyaluronate injections. In more severe cases of knee arthritis, arthroscopic surgery or artificial joint replacement surgery may be necessary.** | 107(14.01) | 456(59.69) | 201(26.31) |
| 1. **In daily life, it's important to wear soft, flexible athletic shoes, choose suitable insoles, and avoid wearing high-heeled shoes.** | 182(23.82) | 463(60.6) | 119(15.58) |
