# Supplementary material for: Knowledge, attitude, and practice towards knee osteoarthritis: a regional study in Chinese patients
Source: Clin Rheumatol. 2025 Mar 11;44(4):1819–30. doi: 10.1007/s10067-025-07385-0 (PMC11993439; doi:10.1007/s10067-025-07385-0)
Supplement: Supplementary file 3 — Supplementary Material 3 (DOCX 15.7 KB) [file 10067_2025_7385_MOESM3_ESM.docx]

**Table S2. Distribution of Attitude Dimension**

|  | **Strongly agree** | **Agree** | **Neutral** | **Disagree** | **Strongly disagree** |
| --- | --- | --- | --- | --- | --- |
| 1. **I hope to receive more science education from healthcare professionals.** | 390(51.05) | 293(38.35) | 57(7.46) | 16(2.09) | 8(1.05) |
| 1. **Joint pain and limited joint mobility make me feel depressed.** | 11(1.44) | 36(4.71) | 163(21.34) | 360(47.12) | 194(25.39) |
| 1. **Long and frequent visits to the hospital without complete relief from knee joint pain or experiencing worsening symptoms have caused me to lose confidence in treatment.** | 14(1.83) | 98(12.83) | 249(32.59) | 292(38.22) | 111(14.53) |
| 1. **To alleviate knee joint pain, I try my best to avoid walking, let alone engaging in other physical exercises.** | 30(3.93) | 262(34.29) | 183(23.95) | 214(28.01) | 75(9.82) |
| 1. **Adjustments to exercise and diet should be made to reduce further damage caused by overweight.** | 172(22.51) | 427(55.89) | 125(16.36) | 37(4.84) | 3(0.39) |
| 1. **To what extent do you agree with the following about barriers to exercise:** |  |  |  |  |  |
| **6.1 Lack of companions for exercise makes it difficult in adhering to exercise.** | 16(2.09) | 167(21.86) | 224(29.32) | 265(34.69) | 92(12.04) |
| **6.2 Concerns about increased pain after exercising.** | 6(0.79) | 121(15.84) | 195(25.52) | 355(46.47) | 87(11.39) |
| **6.3 There's not enough time.** | 11(1.44) | 162(21.2) | 237(31.02) | 286(37.43) | 68(8.9) |
| **6.4 Lack of suitable exercise facilities or venues.** | 10(1.31) | 174(22.77) | 236(30.89) | 271(35.47) | 73(9.55) |
