# Supplementary material for: Knowledge, attitude, and practice towards knee osteoarthritis: a regional study in Chinese patients
Source: Clin Rheumatol. 2025 Mar 11;44(4):1819–30. doi: 10.1007/s10067-025-07385-0 (PMC11993439; doi:10.1007/s10067-025-07385-0)
Supplement: Supplementary file 4 — Supplementary Material 4 (DOCX 16.1 KB) [file 10067_2025_7385_MOESM4_ESM.docx]

**Table S3. Distribution of Practice Dimension**

|  | **Always** | **Often** | **Sometimes** | **Rarely** | **Never** |
| --- | --- | --- | --- | --- | --- |
| 1. **I can strictly follow medical advice. P** | 193(25.26) | 276(36.13) | 230(30.1) | 61(7.98) | 4(0.52) |
| 1. **Daily Life Management** |  |  |  |  |  |
| **2.1 Avoid excessive knee joint activities (such as prolonged walking, running, etc.) P** | 117(15.31) | 260(34.03) | 265(34.69) | 91(11.91) | 31(4.06) |
| **2.2 Reduce climbing stairs to upper and lower floors, prolonged standing, or kneeling positions P** | 120(15.71) | 232(30.37) | 266(34.82) | 116(15.18) | 30(3.93) |
| 1. **Exercise** |  |  |  |  |  |
| **3.1 Swimming P** | 30(3.93) | 50(6.54) | 127(16.62) | 233(30.5) | 324(42.41) |
| **3.2 Perform knee joint flexion and extension exercises without bearing weight P** | 37(4.84) | 99(12.96) | 265(34.69) | 258(33.77) | 105(13.74) |
| **3.3 Consciously contract the quadriceps muscles P** | 26(3.4) | 90(11.78) | 243(31.81) | 257(33.64) | 148(19.37) |
| 1. **Willingness to Accept Therapy** |  |  |  |  |  |
| **4.1 Medication** | 156(20.42) | 227(29.71) | 238(31.15) | 100(13.09) | 43(5.63) |
| **4.2 Orthopedic device therapy** | 364(47.64) | 201(26.31) | 121(15.84) | 49(6.41) | 29(3.8) |
| **4.3 Surgical therapy** | 434(56.81) | 181(23.69) | 95(12.43) | 31(4.06) | 23(3.01) |
| **4.4 Appropriate physical therapy (heat therapy, hydrotherapy, tui na, acupuncture, etc.)** | 150(19.63) | 216(28.27) | 256(33.51) | 103(13.48) | 39(5.1) |
| 1. **I will be proactive in learning disease-related knowledge. P** | 77(10.08) | 187(24.48) | 256(33.51) | 148(19.37) | 96(12.57) |
