# Supplementary material for: Knowledge, attitude, and practice towards knee osteoarthritis: a regional study in Chinese patients
Source: Clin Rheumatol. 2025 Mar 11;44(4):1819–30. doi: 10.1007/s10067-025-07385-0 (PMC11993439; doi:10.1007/s10067-025-07385-0)
Supplement: Supplementary file 5 — Supplementary Material 5 (DOCX 13.8 KB) [file 10067_2025_7385_MOESM5_ESM.docx]

**Table S4. Correlation analysis**

|  | **Knowledge** | **Attitudes** | **Behavior** |
| --- | --- | --- | --- |
| Knowledge | 1 |  |  |
| Attitudes | -0.0515(P=0.1548) | 1 |  |
| Practices | 0.3835(P<0.001) | -0.2287(P<0.001) | 1 |
