# Supplementary material for: Knowledge, attitude, and practice towards knee osteoarthritis: a regional study in Chinese patients
Source: Clin Rheumatol. 2025 Mar 11;44(4):1819–30. doi: 10.1007/s10067-025-07385-0 (PMC11993439; doi:10.1007/s10067-025-07385-0)
Supplement: Supplementary file 6 — Supplementary Material 6 (DOCX 13.8 KB) [file 10067_2025_7385_MOESM6_ESM.docx]

**Table S5. Model fit**

|  | **Ref.** | **Measured results** |
| --- | --- | --- |
| **RMSEA** | <0.08 good | 0.093 |
| **SRMR** | <0.08 good | 0.083 |
| **TLI** | >0.8 good | 0.752 |
| **CFI** | >0.8 good | 0.773 |
