# Supplementary material for: Knowledge, attitude, and practice towards knee osteoarthritis: a regional study in Chinese patients
Source: Clin Rheumatol. 2025 Mar 11;44(4):1819–30. doi: 10.1007/s10067-025-07385-0 (PMC11993439; doi:10.1007/s10067-025-07385-0)
Supplement: Supplementary file 7 — Supplementary Material 7 (DOCX 28.1 KB) [file 10067_2025_7385_MOESM7_ESM.docx]

| Questionnaire No. | |
| --- | --- |
| Dear patient,  We are researchers from the Affiliated Hospital of Binzhou Medical College. We sincerely invite you to participate in our research project. This study aims to understand the patients’ knowledge, attitude and practice towards knee osteoarthritis, to serve as the basis for developing scientific intervention strategies, which may help many others in the future to improve their health conditions. Your participation in this study is voluntary, and the research has been approved by the Ethics Review Committee. If you agree to participate, please read the following instructions:   1. Please complete the questionnaire. There are no right or wrong answers; you only need to provide responses based on your actual experiences. If you have any questions during the process, feel free to reach out to us, and please submit the completed questionnaire in a timely manner. 2. This study is a simple questionnaire survey and will not cause any harm to your physical or psychological well-being. However, it may involve some personal information such as your gender and age. Please rest assured that we will strictly maintain confidentiality and will not disclose your information. 3. As a participant, you can always stay informed about the information and progress related to this study. If you decide to withdraw from the study, please let us know, and your data will not be included in the research results.   Finally, we sincerely thank you for taking the time to support our scientific research amid your busy schedule!  □I have been informed and agreed to the use of the collected data for scientific research.  Informed Consent Signature：  Date of participation： Year Month Day | |
| **Part I Basic Information** | |
| **1.Your gender:** | a.Male  b.Female |
| **2.Your age: .** | |
| **3.Your residence:** | a. Non-urban (rural/ suburban)  b.Urban |
| **4.Your education:** | a.Junior high school and below  b.High school//Technical secondary school  c.Junior college  d.Undergraduate  e.Master and above |
| **5.Your employment:** | a.Full-time  b.Part-time/self-employed/freelancing  c.Unemployed/unemployed/laid off  d.Full-time husband/wife  e.Retired  f.Student  g.Farming |
| 1. **In the past year, your family's monthly per capita income was (including in-kind income and rental income, etc.):______Yuan** | a.<2000  b.2000-5000  c.5000-10000  d.10000-20000  e.>20000 |
| **7.Your marital status:** | a.Unmarried  b.Married  c.Divorced  d.Widowed |
| **8.Do you have a cohabitant?** | a.Yes  b.No |
| **9.Type of medical insurance:** | a.Social medical insurance only (e.g. employee medical insurance, "new agricultural cooperative", "urban residence medical insurance", etc.)  b.Commercial medical insurance only  c.Both of social and commercial medical insurance  d.No medical insurance |
| 1. **Duration of knee osteoarthritis/duration of first consultation: (please select a date).**   If you can't remember the exact dates, please select 1st | |
| **11.Have you ever applied glucosamine hydrochloride or glucosamine sulphate and chondroitin?** | a.Yes  b.No |

| **Part II Knowledge of knee osteoarthritis** | | | |  |
| --- | --- | --- | --- | --- |
| 1. **Knee osteoarthritis is very common, progresses slowly, and gradually develops symptoms over time, sometimes making it impossible to move when it becomes severe.** | a.Known well | b.Heard of | c.Unclear | |
| 1. **The treatment goals for knee osteoarthritis are to alleviate pain, slow down disease progression, correct deformities, improve or restore joint function, and enhance the quality of life.** | a.Known well | b.Heard of | c.Unclear | |
| 1. **Osteoarthritis often occurs in the middle-aged and elderly population, with the prevalence increasing with age, and it may be more common in women than in men.** | a.Known well | b.Heard of | c.Unclear | |
| 1. **Patients with knee osteoarthritis should avoid inappropriate exercise, pay attention to correcting poor posture, refrain from prolonged running, jumping, squatting, and try to avoid climbing stairs for prolonged periods.** | a.Known well | b.Heard of | c.Unclear | |
| 1. **Overweight can increase joint stress, so maintaining a standard body weight is important.** | a.Known well | b.Heard of | c.Unclear | |
| 1. **Engaging in activities like cycling and swimming is beneficial, as they strengthen lower limb muscles, enhance joint stability, and help reduce weight.** | a.Known well | b.Heard of | c.Unclear | |
| 1. **Traditional therapy currently include physical therapy (such as heat therapy, transcutaneous electrical stimulation, and ultrasound), oral medications (glucosamine, nonsteroidal anti-inflammatory drugs, etc.), steroid injections, and sodium hyaluronate injections. In more severe cases of knee arthritis, arthroscopic surgery or artificial joint replacement surgery may be necessary.** | a.Known well | b.Heard of | c.Unclear | |
| 1. **In daily life, it's important to wear soft, flexible athletic shoes, choose suitable insoles, and avoid wearing high-heeled shoes.** | a.Known well | b.Heard of | c.Unclear | |

| **Part III Attitude to knee osteoarthritis** | | | | | |
| --- | --- | --- | --- | --- | --- |
| 1. **I hope to receive more science education from healthcare professionals. P** | a.Strongly agree | b.Agree | c.Neutral | d.Disagree | e.Strongly disagree |
| 1. **Joint pain and limited joint mobility make me feel depressed. N** | a.Strongly agree | b.Agree | c.Neutral | d.Disagree | e.Strongly disagree |
| 1. **Long and frequent visits to the hospital without complete relief from knee joint pain or experiencing worsening symptoms have caused me to lose confidence in treatment. N** | a.Strongly agree | b.Agree | c.Neutral | d.Disagree | e.Strongly disagree |
| 1. **To alleviate knee joint pain, I try my best to avoid walking, let alone engaging in other physical exercises. N** | a.Strongly agree | b.Agree | c.Neutral | d.Disagree | e.Strongly disagree |
| 1. **Adjustments to exercise and diet should be made to reduce further damage caused by overweight. P** | a.Strongly agree | b.Agree | c.Neutral | d.Disagree | e.Strongly disagree |
| **6. To what extent do you agree with the following about barriers to exercise: N** | | | | | |
| **6.1 Lack of companions for exercise makes it difficult in adhering to exercise.** | a.Strongly agree | b.Agree | c.Neutral | d.Disagree | e.Strongly disagree |
| **6.2 Concerns about increased pain after exercising.** | a.Strongly agree | b.Agree | c.Neutral | d.Disagree | e.Strongly disagree |
| **6.3 There's not enough time.** | a.Strongly agree | b.Agree | c.Neutral | d.Disagree | e.Strongly disagree |
| **6.4 Lack of suitable exercise facilities or venues.** | a.Strongly agree | b.Agree | c.Neutral | d.Disagree | e.Strongly disagree |

| **Part IV Practice on knee osteoarthritis**  Always: almost 100% achievable; Often: about 70% achievable or more; Sometimes: about 40-70% achievable; Rarely: about 10-40% achievable; Never: no achievable or less than 10% achievable; | | | | | |
| --- | --- | --- | --- | --- | --- |
| **1. I can strictly follow medical advice. P** | a.Always | b.Often | c.Sometimes | d.Rarely | e.Never |
| **2. Daily Life Management** | | | | | |
| **2.1 Avoid excessive knee joint activities (such as prolonged walking, running, etc.) P** | a.Always | b.Often | c.Sometimes | d.Rarely | e.Never |
| **2.2 Reduce climbing stairs to upper and lower floors, prolonged standing, or kneeling positions P** | a.Always | b.Often | c.Sometimes | d.Rarely | e.Never |
| **3. Exercise** | | | | | |
| **3.1 Swimming P** | a.Always | b.Often | c.Sometimes | d.Rarely | e.Never |
| **3.2 Perform knee joint flexion and extension exercises without bearing weight P** | a.Always | b.Often | c.Sometimes | d.Rarely | e.Never |
| **3.3 Consciously contract the quadriceps muscles P** | a.Always | b.Often | c.Sometimes | d.Rarely | e.Never |
| **4. Willingness to Accept Therapy** | | | | | |
| **4.1 Medication** | a.Very willing | b.Willing | c.Neutral | d.Unwilling | e.Very unwilling |
| **4.2 Orthopedic device therapy** | a.Very willing | b.Willing | c.Neutral | d.Unwilling | e.Very unwilling |
| **4.3 Surgical therapy** | a.Very willing | b.Willing | c.Neutral | d.Unwilling | e.Very unwilling |
| **4.4 Appropriate physical therapy (heat therapy, hydrotherapy, tui na, acupuncture, etc.)** | a.Very willing | b.Willing | c.Neutral | d.Unwilling | e.Very unwilling |
| **5. I will be proactive in learning disease-related knowledge. P** | a.Always | b.Often | c.Sometimes | d.Rarely | e.Never |

| **Thank you again for your participation in completing our questionnaire, the information provided by your answers will be very valuable to us in the future!**  **Thank you for filling out our questionnaire！**  We would be honored to hear from you if you have any comments or suggestions about this research.  Opinions and Suggestions: (optional)  To ensure that this questionnaire is effective and to promote the smooth implementation of future follow-ups, we would be grateful if you could leave your contact details!  Your phone number: (optional) |
| --- |
